# Medical Topography of Green Castle, Indiana, and Vicinity

**Published:** 1848

**Authors:** R. Curran


THE NORTH-WESTERN

### MEDICAL AND SURGICAL JOURNAL.

Vol. I.] OCTOBER AND NOVEMBER, 1848. [No. 4.

### Part 1.—Original Communications.

### ARTICLE I.

### *Medical Topography of Green Castle, Indiana, and Vicinity.*

By R. Curran, M.D.

The town of Green Castle is the site of the Indiana Asbury University, and is situated in the central portion of the State, being the county seat of Putnam county, laying forty miles west of Indianapolis and five miles north of the national road. There are but one or two points in the state, perhaps, more elevated than the spot whereon the town stands. The geological structure of the country around is the same, or nearly the same, as that at the falls of the Ohio at Louisville, but the rocks seem to have a steeper dip, giving a more rapid and easy surface and subterraneous drainage. The knob rock. or that rugged elevation that lies upon the slate, or shale, and approaches the Ohio river, below New Albany, nearly at right angles, extending thence northward towards the interior of the state; as well as the thick stratum of carbonaceous lime-rock which intervenes between it and the coal series, on the banks of the Ohio, seem both to have thinned out before reaching Green Castle, and the coal, commencing in the western edge of Putnam County, seems to lie immediately upon the gray or fossiliferous lime-rock, with here and there interspersed ledges and beds of shale, new red sandstone, and drift.

Owing to the great elevation of this position, the ravines in which run the small water courses are abrupt and steep; they, in their rapid descent, having produced rather grooves in the rocks than basins or beds. The valleys of streams of larger size in the vicinity are very narrow and enclosed by low bluffs, or abrupt banks, cut into sections by deep and sharp ravines. Thus the country is well watered and sufficiently drained, and little liable to inundations from freshets, and, with the exception of some very small spots of table lands, where streams take their rise, nothing is seen to give the least idea of marsh or to produce apprehensions of malaria; still, the country is heavily timbered and exceedingly rich, producing luxuriant growths of succulent plants and weeds, which, in their annual decay, must evolve a certain amount of malaria. The character of the autumnal diseases corresponds with the inferences we would draw from this topographical survey; for, though remittents and intermittents do occur and prevail in the autumnal months, they are not general nor severe. We may attempt to explain another peculiarity in the diseases of this locality, upon this ground—there being no powerful cause to make a strong impression in any particular direction the diseases in this vicinity exhibit a greater variety than is seen in most others equally favorable to health; whatever malarial influence is felt here, seems rather to modify diseased action than to impress any peculiar type. Another peculiarity of this locality may be mentioned, which doubtless exerts some influence in modifying diseased action. That attraction exerted by elevated situations upon the clouds, is well known and is fully illustrated in this situation, this vicinity being frequently visited with gusts of wind and rain, accompanied with an active play of electricity, while surrounding places are exempt; and even in the absence of storms, the atmosphere seems to be in almost constant agitation from currents and counter currents, giving us the winds from almost every point of the compass in the course of twenty-four hours; all of which has a tendency to expedite the evaporation from the surface of the earth, thereby lowering the temperature and increasing the hygrometric moisture.

In such situations the capillary circulation must be disturbed frequently and on occasions violently; but this is abundantly compensated by the purifying effects produced through the agitation of the elements; whatever may be floating there, being precipitated or driven away by the elemental strife. The physical character of the country undergoes but little change by being brought under culture, except what arises from substituting the productions of modern husbandry for those that are natural to the earth in a wild state; this, if any difference, would be rather to the advantage of health. It is the opinion prevalent amongst the oldest inhabitants, however, that disease has been rather on the increase during the past twelve months—if, however, we take the statistics of mortality for the twelve months just past, it presents an aspect very favorable to the salubrity of the locality. The inhabitants of the town of Green Castle may be set down at about two thousand, including two hundred students, the average number in attendance during the past year. The number of deaths in twelve months has been about twenty-five—this gives only one and a quarter per cent. loss in the whole—while three per cent. is the estimated average loss in the population of the whole country. But we must not forget, nor would we suppress the fact, that a larger portion of this loss has been among the students in proportion to their numbers, than among the citizens. Among them there have been six deaths, giving the average of three per cent. among them and reducing it to a small fraction more than one per cent. among the citizens, and for this difference our topographical sketch would not be complete did we not attempt to assign satisfactory reasons. It is a well known fact to all intelligent medical men who have paid any attention to the history of diseases, that epidemics and endemics visit the most favored situations occasionally, carrying alarm and devastation in their course, and then ceasing as suddenly as they appeared, without any seeming cause to produce or give them malignity. From such visitations no locality, prudence, or forecast can shield us; the most healthy situations otherwise, occasionally suffering the most severely. Typhoid fever is one of the forms of disease which in this country has committed the most extensive ravages, and whose course and manner of operating seems to be the most erratic. Though it prevails with most malignity in crowded ships, hospitals, and densely populated cities, yet it occasionally makes its way into the country and spreads all over the country until every other disease wears its livery; the slightest injury any organ or part locally may receive resulting in a fully developed attack of typhoid fever.

This is the form of fever which has prevailed here during the past few months; not indeed in a form so malignant as we have seen it in the country, but still with sufficient severity to create some alarm. The amount of sickness and number of deaths have, however, created more alarm and attracted more attention, in proportion as the thing was unusual and rare before. But the inquiry arises, why did the students suffer more than the citizens? and why did the sickness originate among those who had resided north of the national road?—there having been but little sickness and not a death occurring among those who came from the south part of the state. It may have been owing to the fact that, residing in districts abounding in a more concentrated malaria, their systems may have resisted its power until brought under depressing influences, when it developed disease wearing the livery of that which prevailed for the time, in this vicinity. The largest portion of the sickness was characterized, *ab initio*, with symptoms of adynamia, and this was the more remarkable, having fallen upon young men of firm constitutions and robust make, a large majority of whom had been heretofore accustomed to active life and laborious employments. They seemed to have entered the university, relying upon their strength of constitution, fresh from the duties of life, hard workers, and free feeders.

In the new situation of close student, the bodily labor is suspended but the feeding goes on, sedentary habits within doors are substituted for active employments without. The supply and expenditure being thus disproportioned, the body becomes incumbered with a mass of imperfectly animalized material, having scarcely sufficient vitality to bring it within the control of those physical laws by which it may be appropriated to the supply of the wastes and wants of the system. This want of vitality renders the system incapable of resisting depressing agencies which may be brought to bear upon it. Add to this, the apartments in which most of these young men have slept and spent most of their time, are low, poorly ventilated, and, during the cold weather, heated almost to suffocation with the ever abominable close stove—a room eight by ten, serving for study, dormitory, wood-house, and wash-house;—laying in these places, respiring the same air over and over again, which, by the morning is so much exhausted of its vitalizing properties, especially in that lower stratum where they recline, to which the more impure air naturally sinks, that they arise from their beds rather exhausted than refreshed, and thus they go forth with eagerness into the open air, with systems relaxed, from the double effects of close study and unwholesome situation, and at once encounter the cold damp blasts, upon a skin too susceptible to chill, from the enfeebled circulation, not only upon the skin, but all the free surfaces; from which an impression is at once received disturbing the healthy balance of the circulation, and which imparts a shock, at the same time, to the nervous system, and which being repeated daily, the vital energies under its influence, run down, until the last fragment of vital resistance is gone. This reasoning seems to apply with irresistable force when we reflect, that those who occupied high, airy, and well ventilated apartments, though they did not escape sickness entirely, had disease in a much milder form, and nearly resembling in severity, that which prevailed among the citizens of the place;—with this difference, which, doubtless, arose from the change of habits above alluded to, that the gastric and entiric irritation were much greater. Indeed the mucus lining of the prima via seemed to be the main focus of irritation in most cases; violent and spasmodic pains continuing for a time and followed with obstinate and exhausting diarrhœa, were the usual inceptive stages of a severe attack; and this, when arrested or controlled by the use of the appropriate means, was often followed by exhausting sweats, in which the patient usually sunk with great rapidity. But to enter, at this time, further into the detail of symptoms, would draw us into a deviation from the subject upon which we set out; and as the observations we are making upon the causes of disease in this vicinity, are only in progress, not by any means complete, we shall embody in a future communication some things we had intended for this, if the present, which is extended beyond the original design, should be thought of sufficient importance for your reception and publication.

